# Treatment of a Coronary Bifurcation Lesion Using One Dedicated Sirolimus Eluting Bifurcation Stent in Combination with a Bioresorbable Vascular Scaffold: A Novel Option for Coronary Bifurcation Approach

**DOI:** 10.1155/2016/8402942

**Published:** 2016-03-15

**Authors:** Javier Benezet, Antonio Agarrado, Jesús Oneto

**Affiliations:** Department of Cardiology, Hospital de Jerez de la Frontera, Jerez de la Frontera, Ronda de Circunvalación s/n, 11407 Cádiz, Spain

## Abstract

We present a complex bifurcation lesion treated with a new two-stent strategy combining a dedicated sirolimus eluting bifurcation stent, BiOSS Lim, with a bioresorbable vascular scaffold (BVS). The advantages of this strategy compared with the conventional two-stent approach are as follows: the dedicated stent protects the carina from being damaged, the large cell at the middle zone of the BiOSS Lim gives possibility to enter easily into the side branch (SB) with any standard size conventional device, and, finally, the additional use of BVS in the SB could have a long-term benefit in terms of restenosis.

## 1. Introduction

Coronary bifurcations lesions are considered technically challenging and associated with worse clinical outcomes than nonbifurcation lesions [1]. Although a provisional stenting strategy is the preferred method, a two-stent technique is unavoidable in some settings [2]. Therefore, it is still important to improve two-stent treatment strategies for complex bifurcation lesions in which the side branch (SB) is involved. The use of dedicated bifurcation stents might facilitate the procedure, even in complex and challenging anatomies. The introduction of drug-eluting stents (DES) resulted in a lower event rate and a reduction of main branch (MB) restenosis. However, SB ostial restenosis remains a problem [3]. The use of bioresorbable vascular scaffold (BVS) as SB stent might have a potential benefit after percutaneous coronary intervention (PCI) of bifurcation lesions because, after complete BVS resorption, SB struts can no longer cause late restenosis at the SB ostium as metallic stents do.

We present a case of a bifurcation lesion treated with one dedicated sirolimus eluting bifurcation stent, BiOSS Lim (Balton, Warsaw, Poland) in combination with the Absorb (Abbott Vascular, Santa Clara, CA, USA) BVS. We demonstrate the feasibility of this two-stent technique for the treatment of complex bifurcation lesions.

## 2. Case Report

**Figure 1:**
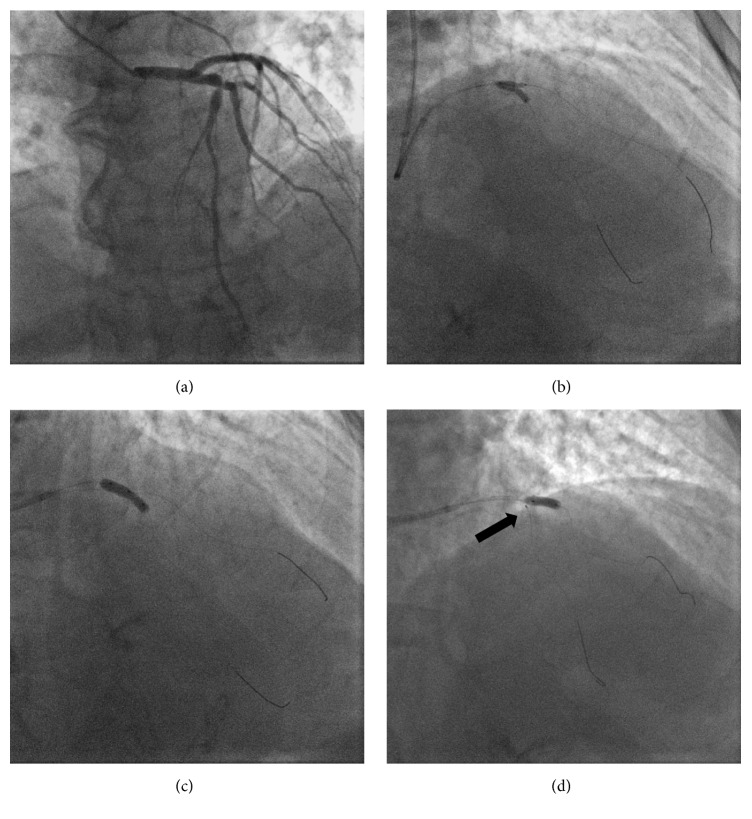
(a) Baseline angiogram. (b) Kissing balloon predilatation. (c) BiOSS Lim sirolimus eluting stent implantation in the proximal-mid left anterior descending artery (main branch). (d) Absorb bioresorbable vascular scaffold implantation in the first diagonal (side branch) with a 1.5 mm balloon in the main branch (“sentinel-balloon technique”) (arrow).

A 59-year-old man with a history of hypertension and hyperlipidemia presented with a 2-month history of exertional chest pain. An echocardiogram showed normal heart wall movement with normal left ventricular ejection fraction and the treadmill test was positive. Diagnostic coronary angiography revealed an 85% stenosis in proximal-mid left anterior descending artery (LAD) and another 80% stenosis in the proximal segment of a large first diagonal (D1) SB (Figure 1(a)) without significant obstructive lesions in the other vessels. That was a Medina class 111 bifurcation lesion [4]. PCI was performed using a right radial artery approach. With a 6 Fr EBU 3.5 guiding catheter the left main coronary artery was engaged. A Sion (Asahi Intecc, Japan) guide wire was placed in the LAD and another one was wired into the D1. A kissing balloon predilatation with a 2.5 × 12 mm semicompliant balloon in LAD and a 2.0 × 12 mm balloon in D1 was done (Figure 1(b)). Then a BiOSS Lim 3.75 mm/3.0 mm × 15 mm stent in direction of LAD was implanted (Figure 1(c)). The BiOSS Lim stent is a coronary, dedicated bifurcation, balloon expandable stent made of 316 L stainless steel with a strut thickness of 120 *μ*m. It is covered with a mixture of a biodegradable polymer and sirolimus. The stent consists of two parts (the proximal one with a larger diameter in relation to the distal one) connected with two struts (average 1.5 mm length) at the middle zone. This zone ensures “self-positioning” of a stent after balloon deflation, as well as the opening to SB. Rewiring or passing other PCI devices through the middle zone of the stent struts becomes simpler and time saving. The BiOSS Lim stent delivery balloon has three markers (distal and proximal indicating stent edges and one midmarker showing the midzone); the midmarker should be placed exactly at the tip of the carina. This ensures that after deployment the contralateral SB wall is covered with struts to the same extent as the proximal MB part of the bifurcation and this can be achieved with routine angiogram [5].

**Figure 2:**
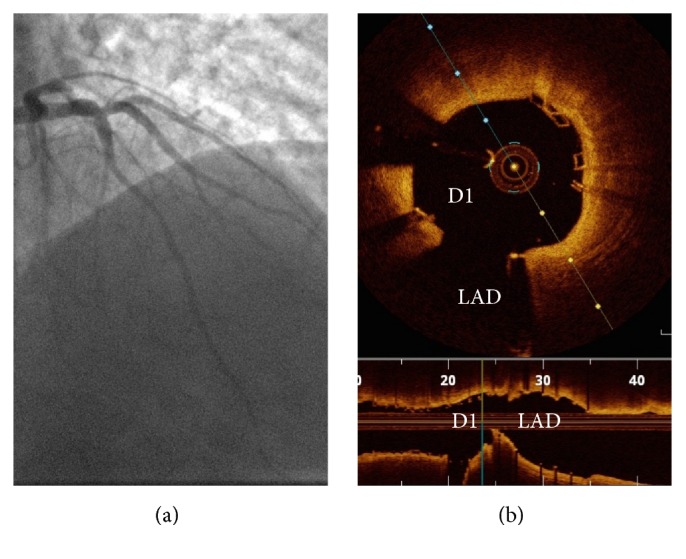
(a) Final angiographic result. (b) Final optical coherence tomography (OCT) from the distal side branch to proximal main branch. Cross section shows the carina with the left anterior descending artery (LAD) and the first diagonal (D1) with the scaffold well apposed.

After BiOSS Lim stent implantation, the D1 stenosis was predilated with a 3 × 8 mm semicompliant balloon that could be easily passed through the BiOSS Lim stent without predilatation to dilate the stent struts. A 3.0 × 12 mm Absorb BVS was positioned through the BiOSS Lim struts into the SB and implanted with the help of a 1.5 × 10 mm balloon (“sentinel-balloon technique”) in the LAD that avoided BVS protrusion into MB. Using as a reference its radiopaque mark, the 1.5 × 10 mm balloon (“sentinel-balloon”) was placed covering the D1 ostium, to guide BVS implantation at the SB ostium (Figure 1(d)). The Absorb scaffold size selection was performed using quantitative coronary angiographic (QCA) analysis. After optimization of the proximal MB with a 4 × 12 mm noncompliant balloon the procedure was finished with final kissing balloon. Final angiography showed no residual stenosis and adequate flow (Figure 2(a)). The QCA analysis revealed that BiOSS Lim and Absorb BVS implantation caused a significant increase of minimal lumen diameter (BiOSS Lim: prestenting 1.88 ± 0.15 mm; poststenting 3.13 ± 0.22 mm; Absorb BVS: prestenting 1.72 ± 0.14 mm; poststenting 2.15 ± 0.16 mm) and decrease of % diameter stenosis (DS) in MB and SB (BiOSS Lim: prestenting 74.2%; poststenting 20,9%; Absorb BVS: prestenting 70.1%; poststenting 20.7%). In the end the bifurcation was assessed by optical coherence tomography (OCT) (C7 Dragonfly, St. Jude Medical Lightlab Inc., Westford, MA, USA) that showed good apposition and expansion of the BiOSS Lim and the Absorb BVS without any scaffold protrusion into MB (Figure 2(b)). Plaque and carina shift (defined as an increase in the plaque-volume and a decrease in the vessel-volume at the SB ostium, resp.) towards LAD were not found after MB stenting. There was no procedural complication and the patient remained angina-free after the procedure and was discharged with 1-year period of dual antiplatelet therapy. After six months of clinical follow-up the patient remained free of angina and cardiac events.

## 3. Discussion

We present a case of complex bifurcation lesion treated with a simple and fast two-stent technique including a single dedicated bifurcation sirolimus eluting stent, BiOSS Lim, for the MB, and an Absorb BVS for the SB. It is an optimal strategy in a complex setting such as the patient of this case report when the SB is stenosed at the ostium over 50% and its diameter exceeds 2 mm.

When the SB is not severely diseased, implantation of a stent in the MB and provisional stenting in the SB is the preferred strategy. But when a two-stent technique is necessary, an effective strategy to stent both branches should be taken as early as possible. An appropriate and timely decision will affect the result, save time and cost, and lower the risk of complications.

Our strategy has several advantages compared with conventional two-stent approach. First, those relating to BiOSS Lim stent: the construction of the BiOSS Lim stent minimizes the negative effects of procedure protecting the carina from being damaged keeping the SB patent without ostium compromise; the large cell at the middle zone of the stent allows an easy access to the SB with any standard size conventional stent and the precision of implantation procedure thanks to the BiOSS Lim three radiopaque markers.

Another interesting detail is the use of BVS in the SB. Using temporary stents eliminates permanent double layers of struts at the SB ostium and could decrease restenosis rates in bifurcation lesions. In addition, the BVS has the potential to restore a more normal vascular physiology of the treated vessel [6]. On the other hand, there are some inherent limitations for using two BVS in two-stent techniques like the difficulty to deliver a BVS to the SB across the MB BVS [7] and the signals for increased early scaffold thrombosis (ST) [8]. The combination of BiOSS Lim stent with a BVS can decrease these limitations. With this novel approach the possibility to use fewer steps than the routine two-stent techniques (less predilatation to allow passage of stents) can save time, contrast, and myocardial ischemic time. About ST, recent studies have raised concern regarding the risk of ST after PCI and implantation of the Absorb BVS [9, 10]. Patients who underwent PCI and placement of a BVS had cardiovascular outcomes comparable to those of patients who received DES but had a higher incidence of definite/probable stent thrombosis. Optimal predilatation and systematic use of intravascular ultrasound or OCT to demonstrate scaffold apposition could reduce the ST rates. Further randomized controlled studies are necessary to assess whether the risk of ST after BVs implantation is increased compared with DES. On the other hand, rates of ST observed early might be offset by a significant reduction in the long-term risks associated with permanent metallic stent covering, although that remains unknown at present.

## 4. Conclusion

To our knowledge, this is the first-in-man combined use of the Absorb BVS and the BiOSS Lim stent. We showed that the combined use of these two devices for the treatment of complex bifurcation lesions was feasible and simplified the procedure.
